# Vasoactive intestinal peptide gene polymorphisms, associated with its serum levels, predict treatment requirements in early rheumatoid arthritis

**DOI:** 10.1038/s41598-018-20400-6

**Published:** 2018-02-01

**Authors:** Iria V. Seoane, Carmen Martínez, Rosario García-Vicuña, Ana M. Ortiz, Yasmina Juarranz, Vanessa C. Talayero, Isidoro González-Álvaro, Rosa P. Gomariz, Amalia Lamana

**Affiliations:** 1 Departamento de Biología Celular, Facultad de Biología, Universidad Complutense de Madrid, Madrid, 28040 Spain; 2 Departamento de Biología Celular, Facultad de Medicina, Universidad Complutense de Madrid, Madrid, 28040 Spain; 3 Servicio de Reumatología, Hospital Universitario de la Princesa, Instituto de Investigación Sanitaria la Princesa, 28006 Madrid, Spain

## Abstract

We previously reported that early arthritis (EA) patients with low vasoactive intestinal peptide (VIP) serum levels demonstrate a worse clinical disease course. In this study, we analysed whether variants in the *VIP* gene correlated with its serum levels and clinical EA parameters. The *VIP* gene was sequenced in patients with extremely high/low VIP levels, measured by enzyme immunoassay. Sixteen single nucleotide polymorphisms (SNPs) were differentially distributed between both groups, which were subsequently genotyped in two patients’ sets. We observed that patients with rs688136 CC genotype showed higher VIP levels in both discovery (n = 91; p = 0.033) and validation populations (n = 131; p = 0.007). This effect was attenuated by the presence of minor alleles rs35643203 and rs12201140, which showed a clear trend towards low VIP level association (p = 0.118 and p = 0.049, respectively). Functional studies with miR-205-5p, which has a target site in the 3′ UTR close to rs688136, revealed a miRNA-mediated regulatory mechanism explaining the higher *VIP* gene expression in homozygous patients. Moreover, patients with an rs688136 CC genotype and no minor alleles of the other polymorphisms required less treatment (p = 0.009). We concluded that the identification of polymorphisms associated with VIP serum levels would complement the clinical assessment of the disease severity in rheumatoid arthritis patients.

## Introduction

Rheumatoid arthritis (RA) is a chronic, polygenic immune-mediated inflammatory disease that leads to significant joint damage, pain, and disability if not treated^1^. The pathogenesis of RA is not completely understood, but genetic factors account for approximately 60% of disease susceptibility^2^, as well as interactions with environmental factors, such as tobacco use and diet, which trigger the development of the disease^3–5^.

RA affects 0.5–1% of the adult population worldwide, resulting in significant social costs in terms of disability, increased comorbidities, impaired quality of life and decreased life expectancy^6,7^.

According to the “window of opportunity” concept, aggressive disease-modifying anti-rheumatic drug (DMARD) treatment at the earliest stages of the disease can prevent irreversible structural damage and even increase the possibility of achieving DMARD-free remission^8,9^. Heterogeneity in the disease course is well-established in RA, therefore treating all patients with aggressive DMARD schedules could be unacceptable in certain cases in which the risk of adverse events could outweigh the benefits. Available biomarkers of severity, such as rheumatoid factor or anti-citrullinated peptide antibodies (ACPAs), do not accurately identify all patients requiring more intensive treatment^10^. Thus, the ongoing search for new prognostic biomarkers represents an important challenge in the management of RA in order to establish an intensive and appropriate treatment at the beginning of the disease, with the purpose of change the disease course in patients predicted to have a worse prognosis. Vasoactive intestinal peptide (VIP) is a homeostatic and immunoregulatory peptide involved in the control of both innate and adaptive immune response. Exogenous administration of VIP exerts therapeutic effects in models of autoimmune/inflammatory disorders, including inflammatory bowel disease^11^, experimental autoimmune encephalomyelitis^12^, Sjögren’s syndrome^13^, and autoimmune diabetes^14^, as well as in a murine model of collagen-induced arthritis^15^. In humans, VIP exerts its protective effects by inhibiting the macrophage proinflammatory polarization profile^16^, changing the Th1/Th2 balance in CD4 T cell differentiation in favor of Th2 cells^17^, promoting the acquisition of a Th17 non-pathogenic profile^18^ and inducing regulatory T cells^18–20^.

The immunoregulatory function of endogenous VIP is supported by the fact that several inflammatory/autoimmune diseases are associated with reduced levels of VIP in serum^21^. In this regard, we have recently reported that patients with Early Arthritis (EA) and early Spondyloarthritis (SpA) who displayed low VIP serum levels at disease onset appeared to develop a greater burden of disease^22,23^. However, using VIP serum levels as a viable biomarker in daily clinical practice presents methodological problems, such as the inter-assay variability of the enzyme immunoassay (EIA) method^22^. Therefore, in this study, we investigated whether variations in the DNA sequence of the *VIP* gene were associated with VIP expression and whether they could reproduce the prognostic value previously described for VIP serum levels. Furthermore, we propose a possible functional mechanism to explain the contribution of one of these genetic variants in the *VIP* gene to its serum levels.

## Results

### Patient characteristics and sequencing of the *VIP* gene

The characteristics of the EA patient subpopulations included in the present study (91 patients for the discovery phase and 131 for the validation phase) are shown in Table 1.

**Table 1:** Baseline characteristics of PEARL subpopulations.

	Discovery population (n = 91)	Validation population (n = 131)	*p*	Meta-analysis (n = 222)
Female gender (%)	64 (70)	108 (82)	0.08	172 (77)
Age	54 [44–66]	54 [44–67]	0.88	54 [44–66]
Diagnosis RA/UA (%)	69 (76)/22 (24)	92 (70)/39 (30)	0.45	161 (73)/61 (27)
Positive RF (%)	38 (42)	73 (56)	0.04	111 (50)
Positive ACPA (%)	42 (46)	70 (53)	0.32	112 (50)
Ever smoker (%)	47 (52)	68 (52)	0.89	115 (48)
DAS28 (0–10)	4.8 [3.5–5.9]	4.1 [3.4–5.5]	0.16	4.5 [3.4–5.7]
HUPI	7.5 [6–10]	6.5 [4.5–10]	0.09	7 [5–10]
HAQ	1 [0.625–1.625]	0.875 [0.375–1.625]	0.29	1 [0.5–1.625]
VIP (pg/ml)	456 [383–501]	529 [460–595]	0.63	453 [384–519]

Data are shown as the median and the interquartile range or as the percentage. RA: rheumatoid arthritis; UA: undifferentiated arthritis; RF: rheumatoid factor; ACPA: anti-citrullinated peptide antibodies; DAS28: 28-joint count Disease Activity Score; HUPI: Hospital Universitario La Princesa Index for disease activity; HAQ: health assessment questionnaire; VIP: vasoactive intestinal peptide. Statistical significance was established by *t* test or Mann-Whitney test for a p-value < 0.05.

We selected 20 patients from the discovery population, which displayed extremely divergent baseline serum VIP levels, for the sequencing screening of the *VIP* gene. The characteristics of this subset of patients are shown in Supplementary Table 1. Sequences and lengths of the primers used for sequencing experiments are detailed in Supplementary Table 2.

### EA patients with high or low serum VIP levels show different genetic variations in the *VIP* gene

**Figure 1:**
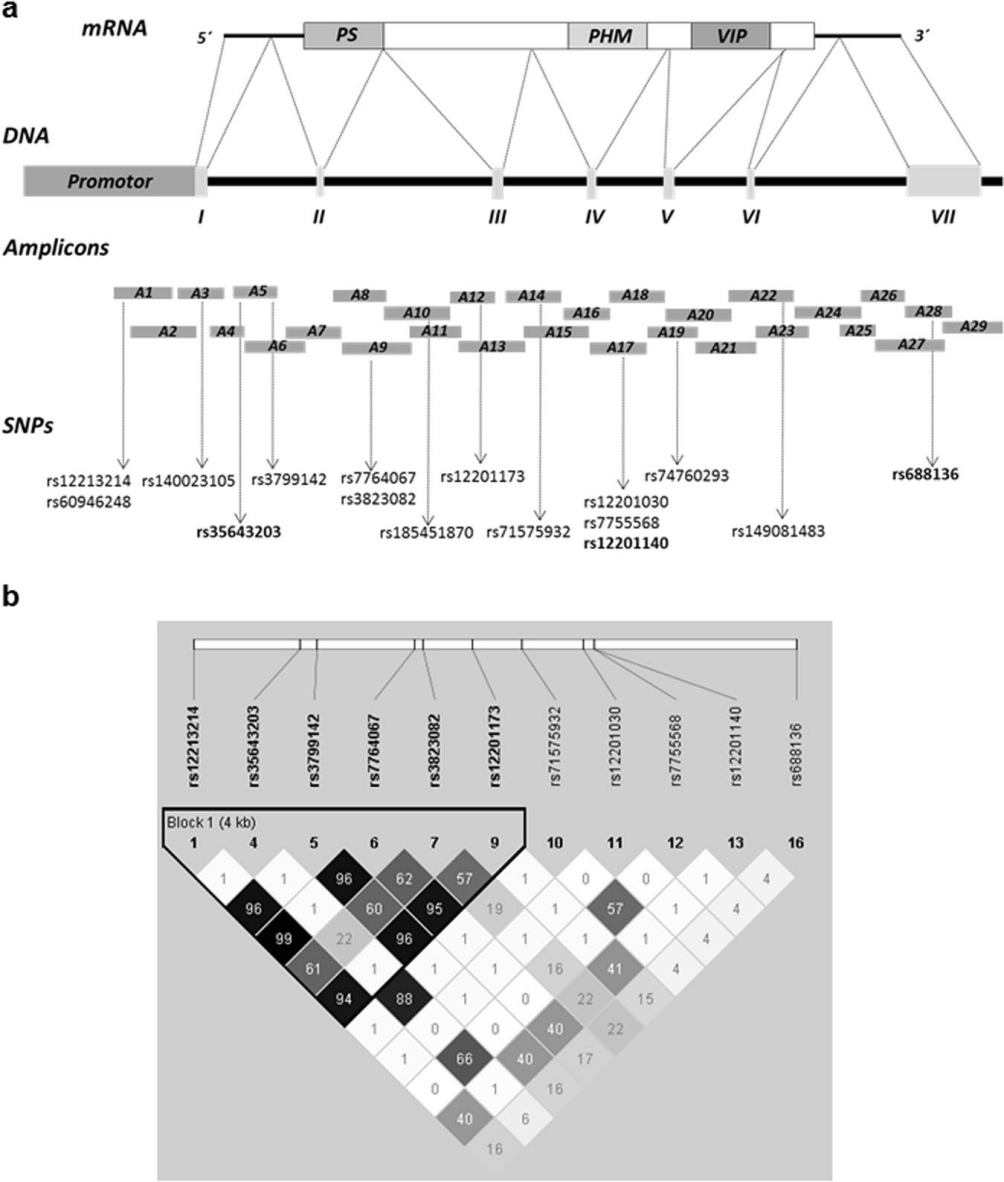
Sequencing of *VIP* gene and linkage disequilibrium (LD) Plot. (**a**) Structural diagram of *VIP* gene comprising the promoter region, 7 exons and 6 introns. The upper diagram shows mRNA transcription and the bottom diagram represents the 29 overlapping amplicons used for sequencing, as well as the SNPs found. SNPs chosen for further study are highlighted in bold. (**b**) Plot showing LD patterns among genotyped SNPs. LD is measured as r^2^ and shown (×100) in the diamond at the intersection of diagonals from each SNP. r^2^ = 0 shown in white, 0 < r^2^ < 1 shown in grey and r^2^ = 1 shown in black. Top analysis track represents chromosomal location. Bold black line outlines one haplotype block designating markers in high LD.

High-quality sequencing data were obtained for 20 patients comprising the screening set. After comparing their *VIP* sequences with the consensus sequence^24^, we found 120 variants, most of which were distributed randomly between the 2 subsets. Among these variants, we observed 16 SNPs differentially distributed in both groups (Fig. 1a and Supplementary Table 3). Next, we genotyped these 16 variants in two different sets of EA patients by sequencing or using SNP TaqMan probes, as detailed in the Methods section. Genotyping of PEARL samples showed that the following five SNPs had a minor allele frequency (MAF) of <5% in our population and were therefore excluded from statistical analysis: rs60946248, rs140023105, rs185451870, rs74760293, and rs149081483 (number of cases and MAF shown in Supplementary Table 3).

Next, we studied whether the rest of the SNPs were in linkage disequilibrium (LD). The R^2^ value was >0.9 for rs12213214, rs3799142, rs7764067 and rs12201173. We chose rs12213214 as the tagSNP for this group since that polymorphism is located in the promoter region. In addition, rs3823082 showed an R^2^ = 0.614 with rs12213214. As shown in Fig. 1b, applying the Gabriel method^25^, we observed that these five SNPs formed a haplotype block. On the other hand, rs35643203 and rs71575932 were in LD (R^2^ = 0.88), and we chose rs35643203 as the tagSNP. The remaining polymorphisms were studied separately since they were not in LD with any other polymorphism.

### Genetic variants in the *VIP* gene influence serum levels of VIP in EA patients in the discovery phase

Next, to assess the relationship between VIP levels and selected polymorphisms on the *VIP* gene, we performed a preliminary bivariate analysis. As shown in Supplementary Table 4, in the discovery population, patients with one minor allele of rs35643203 had significantly lower serum VIP levels than homozygous patients for the major allele. In addition, patients carrying one minor allele of rs3823082 and rs7755568 showed a non-significant trend towards lower VIP levels than those with two copies of the major allele. On the other hand, the only SNP that appeared to be related with increased VIP levels was rs688136. For this reason, we decided to include it in the multivariable analysis.

We have previously described that there were no significant differences in VIP serum levels between healthy donors and patients with arthritis. However, more heterogeneous ranges of VIP serum levels were observed in patients with either RA or UA (Undifferentiated Arthritis)^22^. In addition, age at disease onset and use of TNF-blocking agents has been reported to influence VIP levels^22,23^. Therefore, we decided to perform a multivariable analysis to further elucidate the precise contribution of these SNPs to the evolution of serum VIP levels during the course of arthritis.

In this context, and according to what we have described before, we observed that in patients who were treated with TNF blockers, VIP serum levels were significantly increased (Supplementary Table 5; Model 1). In addition, we confirmed that older patients tended to show higher VIP levels (Supplementary Table 5; Model 1). After adjusting for these confounders, we found that patients with the CC genotype of the rs688136 allele had significantly higher levels of serum VIP (Supplementary Table 5; Model 1; p = 0.033). In contrast, having one minor allele of rs35643203 or of rs12201140 were associated with lower serum VIP levels (Supplementary Table 5; Model 1; (p = 0.05) and (p = 0.068), respectively).

### Validation phase and meta-analysis

To confirm the association between SNPs of the *VIP* gene and VIP serum levels observed in the discovery population, we replicated the analyses in a new set of 131 patients. The association between CC genotype for rs688136 with higher VIP levels was confirmed in both bivariate analysis (p = 0.002) (Supplementary Table 4) and multivariate analysis (p = 0.007) (Supplementary Table 5 model 1).

**Figure 2:**
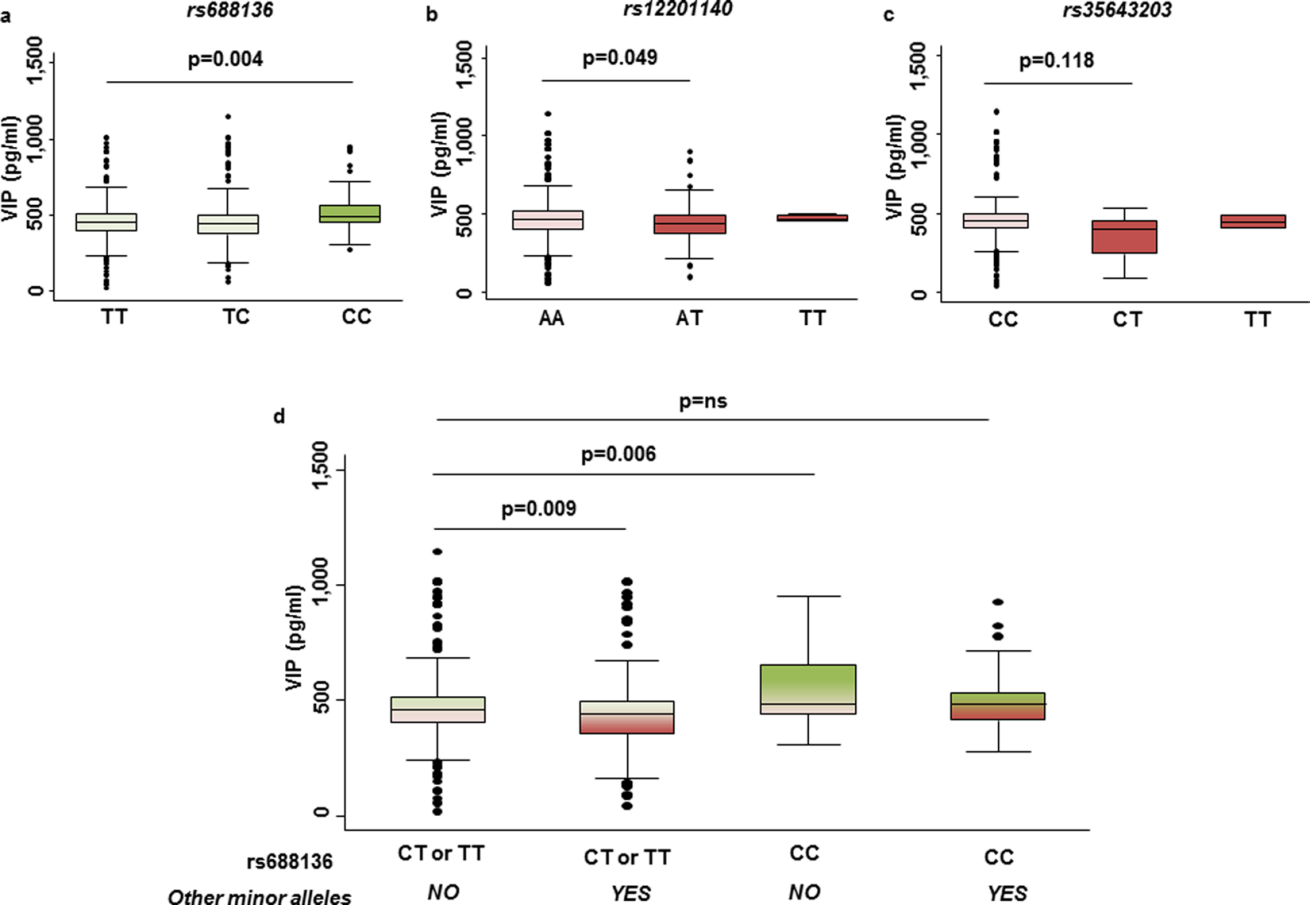
Serum VIP levels in EA patients according to different genotypes. Serum VIP levels according to the genotype of the: (**a**) rs688136; (**b**) rs12201140; (**c**) rs35643203. (**d**) Interaction Score for different genotype combinations of *VIP* gene polymorphisms associated with VIP serum levels. In all panels data from the meta-analysis (n = 222) are presented as the interquartile range (p75 upper edge of the box, p25 bottom edge, and p50 midline), p90 and p10 (lines below and above the box) of the serum VIP levels. Statistical significance for a p-value < 0.05 was established by means of a multivariate analysis adjusted by age of onset and TNFα blockers and nested by patient and visit. Dots represent outliers. ns: non-significant.

In a further step, a meta-analysis of both populations (n = 222) performed for rs688136 showed that the association between CC genotype and high VIP levels remained statistically significant, both in the bivariate (p = 0.008) and multivariate analysis (p = 0.004) (Fig. 2a, Table 2 model 1 and Supplementary Table 5). By means of the multivariate analysis, we also confirmed a significant association between serum VIP levels and rs12201140 in the meta-analysis (p = 0.049) (Fig. 2b and Table 2 model 1) and a non-significant trend for the rs35643203 (p = 0.118) (Fig. 2c and Table 2 model 1).

**Table 2:** Polymorphisms associated with VIP serum levels during the follow-up of patients with early arthritis in a multivariate analysis.

	Model 1	*p*	Model 2	*p*
	β coeff ± SE		β coeff ± SE	
rs688136				
TT	Ref.		—	—
TC	−1 ± 16.1	0.929		
CC	79 ± 27.9	0.004		
rs35643203				
CC	Ref.		—	—
CT	−38 ± 24.1	0.118		
TT	16 ± 81.3	0.845		
rs12201140				
AA	Ref.		—	—
AT	−35 ± 17.6	0.049		
TT	−100 ± 71.3	0.162		
Interaction Score				
CT or TT and no other minor alleles	—	—	Ref.	
CT or TT and other minor alleles			−37 ± 14.1	0.009
CC and no other minor alleles			99 ± 36.3	0.006
CC and other minor alleles			12 ± 27.8	0.669
Onset Age				
<45	Ref.		Ref.	
45–65	31 ± 18.4	0.090	31 ± 16.2	0.052
>65	27 ± 20.3	0.179	33 ± 17.7	0.065
TNF blockers				
No	Ref.		Ref.	
Yes	73 ± 34.1	0.031	65 ± 30.8	0.036

The longitudinal analysis was performed with data from consecutive visits corresponding to the meta-analysis (n = 222). Included patients had, at least, two visits along the follow-up. Signification was established by means of generalized estimating equations nested by patient and visit. Multivariable model fitted by a backward-stepwise selection. Model 1 fitted including independent polymorphisms and Model 2 including the Interaction Score. Ref.: reference value; β coeff: beta coefficient; SE: standard error; TNF: tumour necrosis factor.

### Interactions between polymorphisms in the *VIP* gene could modify VIP serum levels

Since we observed a marked heterogeneity in VIP serum levels among genotypes of each SNP, we investigated if interactions between the genetic variants of the *VIP* gene could explain this variability. Thus, we created an Interaction Score as a categorical variable that let us cluster the patients into 4 groups according to the different genotype combinations of the polymorphisms rs688136, rs35643203 and rs12201140. Clusters were established as follows:

- Patients carrying the CT or TT genotype of rs688136 and no rs35643203 or rs12201140 minor alleles.
- Patients carrying the CT or TT genotype of rs688136 and at least 1 minor allele of either rs35643203 or rs12201140.
- Patients carrying the CC genotype of rs688136 and no rs35643203 or rs12201140 minor alleles.
- Patients carrying the CC genotype of rs688136 and at least 1 minor allele of either rs35643203 or rs12201140.

We consider the population of reference to be the one that showed the minimum variations with respect to the consensus sequence in relation to the genetic variants of the study, that is, the population of patients with CT or TT genotypes of rs688136 and no minor alleles of the other polymorphisms. As shown in Fig. 2d, patients with a CC genotype of rs688136 and no minor alleles of the other SNPs showed the highest VIP serum levels (p = 0.006). The group with the lowest levels was the one who had at least one major allele of rs688136 and at least one minor allele of the other SNPs (Fig. 2d, Table 2 model 2). Interestingly, those patients with the CC genotype of rs688136 (associated with high VIP levels) and at least,one minor allele of rs35643203 or rs12201140 (associated with low VIP levels) displayed similar VIP levels to the reference population (Fig. 2d and Table 2 model 2).

### Minor allele of rs688136 interferes with the downregulation of *VIP* expression induced by miR-205-5p

**Figure 3:**
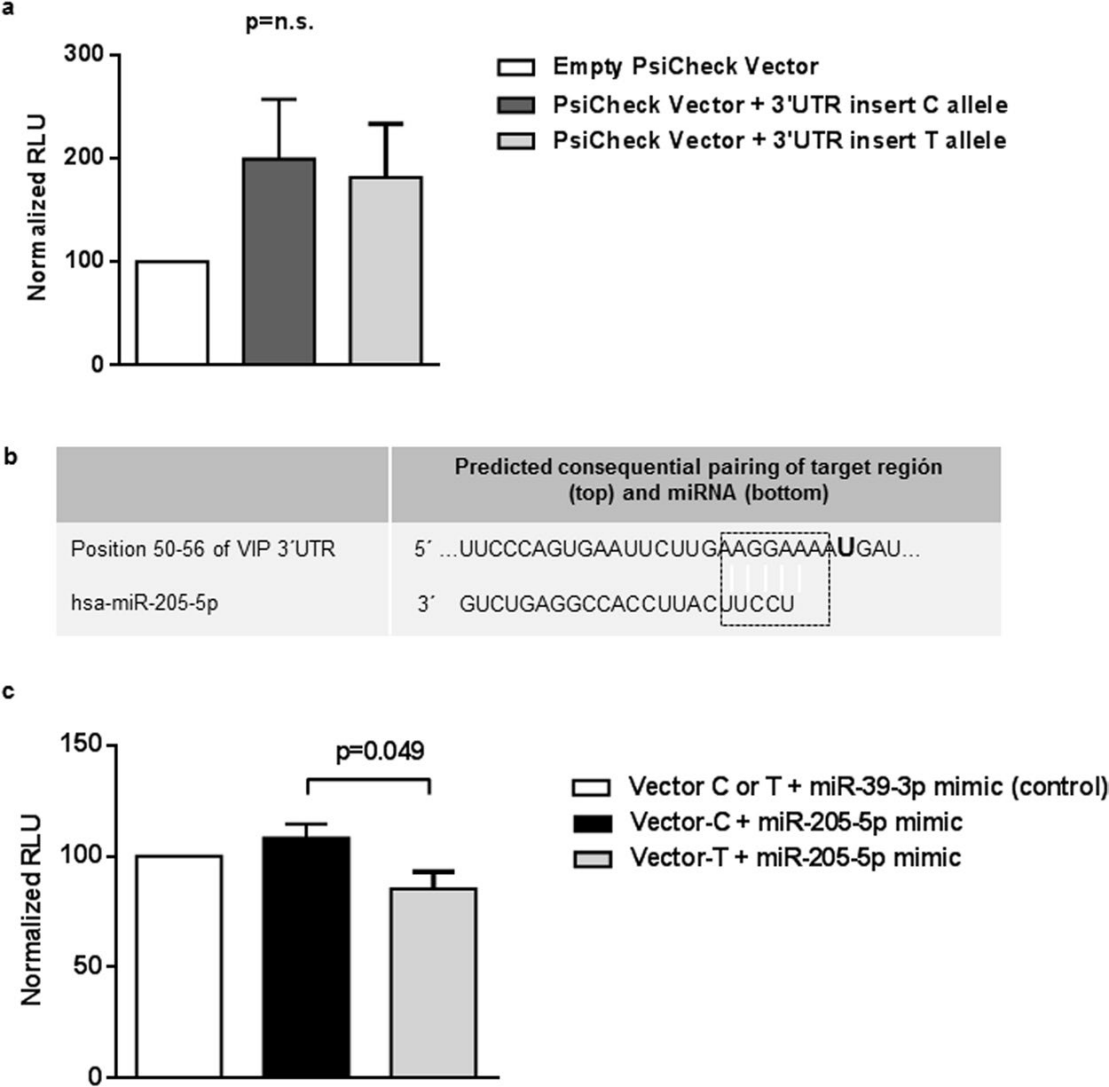
VIP 3′UTR as a target of miRNA miR-205-5p. (**a**) Transfection with empty PsiCheck2 vector and PsiCheck2 vector-3′UTR inserts with T or C allele for the rs688136 respectively. Data are represented as the mean ± SEM. n = 10 each group. n.s: non-significant. (**b**) Bioinformatic prediction of hsa-miR-205-5p recognition elements on human *VIP* 3′UTR. Analyses performed with the TargetScanHuman algorithm. Letter U highlighted in bold corresponds to rs688136 position. Dotted box indicates pairing region. (**c**) Co-transfection with PsiCheck2 vector-3′UTR inserts with T or C allele and mimic miRNAs miR-205-5p and miR-39-3p (control). Data are represented as the mean ± SEM. n = 8 each group. RLU: relative luminescence unit. Statistical significance in panels a and c was established using Mann-Whitney test for a p-value < 0.05. Each experiment was made in duplicate.

Next, we tried to elucidate the mechanism involved in the differences of VIP serum levels associated with the presence of the T/C allele of the rs688136. Since this polymorphism is located at the 3′UTR, which plays a role in the regulation of the expression of most mRNA, the PsiCheck-2 vector was constructed as explained in the Methods section. A Dual Luciferase Assay was performed in order to determine whether the mere presence of the T allele was associated with a minor luciferase activity and, thus, to a reduced gene expression. As shown in Fig. 3a, we did not observe statistically significant differences between the luciferase activities of both vector constructs, but there was a trend towards a decreased expression in the presence of the T allele.

As miRNA binding sites have been described as one of the regulatory mechanisms in the 3′UTR, we searched in Target Scan (www.targetscan.org) and miRBase (www.mirbase.org) for miRNAs with predicted binding sites close to the SNP rs688136 and identified the has-miR-205-5p (Fig. 3b). Thus, we co-transfected Jurkat cells with the PsiCheck-2 vectors (T-insert or C-insert), and this miRNA. Another co-transfection with the mimic cel-miR-39-3p was performed as a control miRNA experiment. The result of the Dual Luciferase Assay showed that in the presence of miR205-5p, cells co-transfected with the T-insert vector displayed a significant decreased gene expression in terms of lower luciferase activity, whereas the vector with the C allele of rs688136 was resistant to the regulatory effect of miR-205-5p (Fig. 3c; p = 0.049).

### Combinations of genetic variants of *VIP* gene are associated with treatment requirements in EA patients

Finally, as we have previously reported that low serum VIP levels are associated with poor outcomes in EA patients^22^, we investigated whether genetic variants of *VIP* associated with its protein levels were also associated with disease severity. No relevant differences in disease activity were observed among patients with different genetic variants of *VIP* (data not shown), likely due to a wide implementation of the *treat-to-target* strategy in our population^26^. Therefore, we studied the intensity of DMARD treatment as a surrogate variable of disease severity. As described previously by our group and others^10^, female patients and ACPA-positive patients showed a trend towards more intense DMARD treatment (Table 3). In contrast, patients older than 65 years old and those not fulfilling the RA criteria received less intense treatment (Table 3). Finally, as is well-known, a significant variability in the intensity of DMARD treatment among physicians was observed^10,27^ (Table 3). Adjusting for these variables, carrying the CC genotype of rs688136 and no other minor alleles of rs35643203 and/or rs12201140 was significantly associated with less intensive treatment requirements, whereas carrying the CC genotype plus at least one of the minor alleles of the SNPs associated with lower VIP levels (Table 3, p = 0.009; Supplementary Table 6). Therefore, similar to our previous description of VIP serum levels^22^, the genotype associated with high VIP levels was also associated with less severe disease and thus with a reduced need for intensive treatment.

**Table 3:** Association of genotype combinations of the Interaction Score with the Intensity of Treatment in patients with early arthritis. Multivariable analysis.

	β coeff ± SE	*p*
Interaction Score		
CT or TT and no other minor alleles	Ref.	
CT or TT and other minor alleles	−0.14 ± 0.09	0.131
CC and no other minor alleles	−0.62 ± 0.24	0.009
CC and other minor alleles	−0.04 ± 0.19	0.849
Gender		
Male	Ref.	
Female	0.15 ± 0.11	0.174
OnsetAge		
<45	Ref.	
45–65	−0.01 ± 0.11	0.958
>65	−0.11 ± 0.12	0.363
Diagnosis		
RA	Ref.	
UA	−0.85 ± 0.11	0.000
ACPA		
Negative	Ref.	
Positive	0.12 ± 0.10	0.226
Physician		
0	Ref.	
1	0.09 ± 0.18	0.660
2	0.69 ± 0.14	0.000
3	−0.41 ± 0.24	0.119
4	0.06 ± 0.14	0.621
5	0.31 ± 0.16	0.042
6	0.32 ± 0.13	0.007

Association analysis performed with data corresponding to the meta-analysis (n = 222). Signification was established by means of generalized estimating equations. Multivariable model fitted by a backward-stepwise selection. Ref.: reference value; β coeff: beta coefficient; SE: standard error.

## Discussion

Studies of genetic variants influencing the risk of developing autoimmune diseases are numerous^28^. However, there are few reports concerning how genetic variability can modulate the severity of autoimmune disorders. This latter approach may be particularly useful when designing an optimal treatment for patients with autoimmune diseases. In this regard, to our knowledge, this study is the first to demonstrate that several polymorphisms of the *VIP* gene are associated with differences in serum levels in patients with early arthritis. Considering that low serum VIP levels have been associated with worse disease courses and greater treatment needs^22^, the SNPs of *VIP* identified herein and associated with VIP serum levels could be potential indicators of treatment requirements and, therefore, disease severity in patients with recent onset.

VIP is an endogenous mediator involved in the maintenance of homeostasis of the endocrine, nervous, and immune systems^29–31^. Administration of exogenous VIP has improved clinical outcomes in several experimental inflammatory/autoimmune murine models^11–15,32^. Moreover, *ex vivo* assays with synovial fibroblasts (SFs) and peripheral blood lymphocytes (PBLs) in RA patients have supported these results. VIP is able to reduce the production of inflammatory mediators by SFs via TLR signalling pathways^33–35^ and modify the functional capacity of human lymphocytes by inducing Th2/Treg differentiation^36^. In light of this collective evidence, it can be hypothesized that low serum VIP levels may lead to inadequate control of the immune system. In this sense, decreased levels of VIP have been described in patients with autoimmune diseases, such as multiple sclerosis^37^ and autoimmune thyroiditis, in this case associated with high levels of VIPase autoantibodies^38^. Moreover, reduced levels of VIP are associated with more severe disease in Chagas cardiomyopathy^39^ and juvenile idiopathic arthritis, with clinical evidence of cardiac autonomic neuropathy^40^. In rheumatic diseases, an association between low VIP levels in serum and worse outcomes in patients with RA and SpA^22,23^ and joint damage in osteoarthritis patients has been reported^41,42^.

Our results show that variations of the *VIP* gene affect its serum levels in EA patients. Specifically, we reported that rs35643203 and rs12201140, which are located in intron regions 1 and 4, respectively (Fig. 1a), are associated with a lower concentration of VIP in serum. The mechanisms through which those SNPs regulate the expression of VIP levels remains unclear, but several SNPs located in intronic regions of genes such as *STAT4* and *CLEC16A* have been reported to modify the expression of their mRNA and confer a higher risk of developing RA^43,44^. It has been suggested that the presence of SNPs in intronic regions could affect splicing efficiency by generating a transcript that lacks an exon, as reported for the *HMGCR* gene^45,46^, or by altering the stability of mRNA, as described for the *MDR1* gene^47^. However, the functional significance of the SNPs herein remain to be elucidated.

In contrast, rs688136, located at exon 7 encoding for the 3′UTR region of the mRNA, was associated with increased levels of VIP. Since the 3′UTR region can include regulatory sequences, polymorphisms in this area could elude regulatory mechanisms, thereby resulting in the modification of VIP expression. One of these mechanisms are miRNAs, small endogenous RNA species that primarily target the 3′UTR of mRNA transcripts with well-known roles in suppressing translation or inducing degradation of mRNA^48,49^, consequently modulating gene expression. A putative target sequence of miR-205-5p was identified in the *VIP* 3′UTR near rs688136. Our data suggest that the C allelic variant in that SNP is not recognized as efficiently as the T variant by miR-205-5p, resulting in increased VIP mRNA expression and in high serum levels of this peptide. There are indeed several studies showing how the presence of SNPs in 3′UTR can affect microRNA binding and, thus, its regulatory effects on gene expression^50–52^.

We also contributed to the development of a variable that allowed us to assess the effect of genotype combinations associated with serum VIP levels, the *Interaction Score*. This variable could potentially correlate with clinical outcomes, as we have previously described for serum levels^22^.

Our study is subject to several limitations. First, and not surprisingly, serum VIP levels cannot be thoroughly explained by the presence of the genetic variants described herein. Consequently, other mechanisms must be involved in the modulation of serum VIP levels. It is necessary to perform more in-depth studies regarding other regulatory mechanisms recently described in RA, such as epigenetic changes. Second, our data set comes from a single cohort of patients. However, it should be noted that a replication of our results was performed in two independent subpopulations separated in time by more than a year. Moreover, the longitudinal design enabled us to evaluate a considerable number of observations and to better control individual variations. Third, the intensity of treatment is a time-dependent variable and is therefore affected by changes in therapeutic strategies and physicians. To overcome this limitation, all our analyses have been properly adjusted. Additionally, we have a cohort in which the performance criteria are homogeneous and a complete, rigorous and exhaustive collection of data concerning treatment with DMARDs was accomplished throughout the follow-up.

Conversely, it is well-known that severity assessment in RA patients is highly difficult because it is a compendium of biological, social and psychological factors. Moreover, as arthritis is a multifactorial disease, the classical outcomes do not always show the expected efficacy. In this sense, there is an urgent need for treatment as a pertinent surrogate marker of disease severity in early arthritis cohorts^10^.

In summary, we demonstrate an association between serum VIP levels and variants in the *VIP* gene in EA patients. Functional findings suggest the potential involvement of the 3′UTR region in the modulation of *VIP* gene expression, possibly through a miRNA-mediated mechanism. We also describe an interaction between different genetic variants and how certain combinations of genotypes of these variants are associated with a higher intensity of treatment in our patients.

Taken together, these results underscore the importance of the detection of VIP genetic variants that enable EA patients to be stratified for therapeutic decision making in the ‘window of opportunity’ in EA. In this regard, it is likely that our results suggest that ACPA-negative patients carrying the CC genotype of rs688136, without minor alleles of rs35643203 or rs12201140, will be candidates for less intensive therapy.

## Patients and Methods

### Study population

The Princesa Early Arthritis Register Longitudinal (PEARL) study comprised patients referred to the Early Arthritis Clinic at the Hospital Universitario La Princesa, Madrid (Spain). To be referred to the clinic, patients must have had one or more swollen joints for less than a year. Patients with other specific causes of arthritis were excluded. The register’s protocol included five visits during a follow-up period of two years (baseline, 6, 12, 24 and 60 months). At each visit, the following data were collected and entered into an electronic database: clinical and demographic information; disease duration at the beginning of the follow-up; 28 tender and swollen joint counts (TJC and SJC, respectively); global disease activity on a 100 mm visual analogue scale assessed both by the patient (GDAP) and the physician (GDAPh); Spanish version of the Health Assessment Questionnaire^53^ and laboratory tests including erythrocyte sedimentation rate (ESR), C-reactive protein (CRP) and RF levels assessed by nephelometry (positive >20 UI/ ml) and ACPA measured by enzyme immune assay (EIA) (Euro-Diagnostica Immunoscan RA; positive >50 UI/ml). A more detailed description of the PEARL protocol has been published previously^54^.

For this study, we analysed data from 845 visits belonging to two sets of patients: the discovery phase included 91 patients enrolled from 2001 to 2009 (349 visits), and the validation phase included 131 patients enrolled from 2006 to 2013 for (496 visits). The characteristics of these subpopulations of PEARL are shown in Table 1. To be included in the study, patients must have had at least 2 years of follow-up, either those who met the 1987 American College of Rheumatology criteria for RA^55^ or those considered to have undifferentiated arthritis (UA)^56^, since other specific diagnoses were ruled out. During the 1980s, the term “undifferentiated arthritis” had been used to refer to a subpopulation of patients who falls within the spectrum of spondyloarthritis. However, during the first decade of this century, UA was more commonly used to refer to a disease that could be considered a pre-RA disorder in a considerable proportion of patients^56–59^.

The Instituto de Investigación Sanitaria La Princesa Research Ethics Committee reviewed and approved the protocol of PEARL study (PI-518), and all experiments were performed in accordance with the guidelines and regulations of this committee. All patients signed an informed consent form before data were included in the register, and biological samples were stored at the local Biobank.

### Measurement of serum VIP levels

VIP levels were assessed using a commercially available competitive enzyme immunoassay kit (Phoenix Pharmaceuticals, Karlsruhe, Germany) according to the manufacturer’s instructions. To avoid the effects of inter-assay variability of this test in the measurements of each patient, all samples corresponding to consecutive visits from the same patient were measured together on the same plate. The intra-assay and inter-assay variation coefficients were ≤5% and 15%, respectively, as previously described^22^.

### Genetic studies

#### Screening DNA sequencing

Genomic DNA was isolated from whole blood using the QIAamp DNA Blood Midi Kit (QIAGEN, Hilden, Germany). We selected 20 patients from the discovery population who displayed extremely divergent baseline serum VIP levels: 11 of them had serum VIP levels above 601 pg/ml, and the remaining 9 patients had serum VIP levels below 272 pg/ml [p75 and p25 of a control population, respectively^22^]. The characteristics of these PEARL subpopulations used for the sequencing analysis are shown in Supplementary Table 1.

Amplification primers were designed to produce overlapping PCR amplicons to cover the region between positions 153071932 and 153080900 (GRCh37.p13) of chromosome 6, which includes the promoter and the gene encoding VIP. A total of 58 PCR primer pairs were designed, including the M13 universal primer sequence (M13 forward primer sequence, 5′- TGTAAAACGACGGCCAGT-3′; and M13 reverse primer sequence, 5′- CAGGAAACAGCTATGACC-3′) to generate 29 amplicons ranging between 393 bp and 788 bp (Fig. 1a and Supplementary Table 2). Amplification and cycle sequencing were performed using the BigDye Direct Cycle Sequencing Kit, purified using the BigDyeXTerminator Purification Kit and sequencing by 3500xLGenetic Analyzer (Applied Biosystems).

#### SNP genotyping

Genetic variants selected in the sequencing screening were genotyped in 91 and 131 EA patients from PEARL for discovery and validation phases of the study, respectively.

The selected *VIP* genetic variants rs12213214, rs140023105, rs35643203, rs3799142, rs7764067, rs3823082, rs12201173, rs71575932, rs74760293, rs149081483 and rs688136 were genotyped using a pre-designed single nucleotide polymorphism (SNP) Genotyping Assays (Part numbers: C__27847302_10, C__27855145_10, C___3250637_10, C__27502877_20, C__29430252_10, C__27491244_10, C__32237894_10, C___3250638_10, C__25962626_10, C_172567893_10, C___3250639_10, respectively), and rs60946248 and rs185451870 were genotyped using a custom SNP Genotyping Assay (Applied Biosystems).

After PCR, the genotype of each sample was determined automatically by measuring allele-specific fluorescence on a CFX Touch Real-Time PCR System using the software CFX 3.1 Manager (BioRad). Duplicate samples and negative controls were included to verify genotyping accuracy.

The genotype of rs12201030, rs7755568 and rs12201140 was obtained by sequencing amplicon 17, as we described above (see amplicon information in Supplementary Table 2). These SNPs are located in a low complexity region with a predominance of A and T bases, making it impossible to obtain specific TaqMan probes.

#### Generation of VIP 3′UTR luciferase reporter vectors

Neuroblastoma cell line SH-SY5Y was used to obtain the VIP 3′ untranslated region (3′UTR), where rs688136 is located. That region was amplified by means of PCR using forward and reverse custom primers with the restriction sites for XhoI and NotI added (primer forward 5′-TGAAAAAGACCTTTGGAGCAAAGCTGATGACAA-3′, primer reverse, 5′-CAGGAGAGTAGAACAGATAATCAGTGTGTCTAAATTTG-3′). The PCR amplified 3′UTR 333 bp region was cloned into the PsiCheck-2 vector (Promega), in which two different luciferase genes (Firefly and Renilla) driven by separate promoters are present. VIP 3′UTR inserts (containing T or C alleles for the rs688136) were cloned following the Renilla luciferase gene, which served as the reporter, while the Firefly luciferase gene was the internal control.

Transformation was performed in competent bacteria, and transformed colonies were selected by culturing in the presence of Ampicillin.

#### Transfection and luciferase assay

Jurkat cells were grown to a 10^6^/ml confluence in complete media (RPMI, 10% FBS and 1% Penicillin/Streptomycin). Next, 10^7^cells were electroporated with 20 µg of the PsiCheck-2 vector constructs (alleles T or C) and, in the co-transfection experiments, 2 pmol of miRCURY LNA microRNA Mimics (Exiqon), cel-miR-39-3p (microRNA Mimic control) and has-miR-205-5p. Positive controls containing the empty PsiCheck-2 vector were included. After culturing for 24 h at 37 °C, transfected cells were lysed in 500 µl 1X passive lysis buffer (Promega). Lysates were used in each dual luciferase reaction, conducted according to the manufacturer’s instructions (Dual-Luciferase Reporter Assay System, Promega). Triplicate Renilla measurements were performed using FLUOstar Omega luminometer (BMG Labtech) and normalized to Firefly measurements. Luminescence measurements of has-miR-205-5p co-transfected cells were normalized to the corresponding measurements of cel-miR-39-3p co-transfected cells.

### Statistical analysis

Normally distributed quantitative variables were expressed as the means ± standard deviation, while non-normally distributed variables were expressed as the median and interquartile range (IQR). In cases of Normal distribution, bivariate analyses were performed using the *t* test, while Mann-Whitney or Kruskal-Wallis test were used for non-normal variables.

Additional variables were defined to further describe the impact of *VIP* genetic variants in VIP protein serum levels and in the course of arthritis. The variable *Interaction Score* was developed as a measure of the effect of the combination of different genotypes, clustering patients into four categories. On the other hand, the implementation of the early “treat to target” EULAR recommendation strategy in PEARL patients has yielded similar disease activity outcomes, rates of remission and decreases of radiological progression in both ACPA-positive and -negative patients. Therefore, we decided to use the intensity of DMARD treatment (IDT)^60^ as a surrogate variable of severity to study whether genetic variants in VIP could be associated with severity in early arthritis patients. IDT was assessed as the number of days of treatment with each DMARD during follow-up adjusted for weighted coefficients, as described previously^54^.

Serum VIP levels showed a distribution that was almost normal in both sets of patients. On average, we had measurements of at least 3 samples per patient, corresponding to different follow-up visits, in order to minimize the intra-assay variability. Nevertheless, we had previously reported that VIP serum levels do not vary relevantly during the follow-up^22^. However, several variables may contribute to slight modifications in VIP levels as well as they can interact with the effect of variants of *VIP* gene on these levels, so we performed several multivariate analyses based on generalized estimating equations nested by patient and visit using the *xtgee* command of Stata 12 for Windows (Stata Corp LP, College Station, Texas, USA). This model allows us to achieve a better adjusted VIP value for each patient. First, we performed the multivariate models by adding all variables with a *p* value <0.15 in the bivariate analysis. Then, we used the manual backward-stepwise selection to fit the final models by sequentially removing variables with *p* > 0.15. Multivariable models were in all cases adjusted by the variable “assay plate” in order to minimize the inter-assay variability. Since the intensity of treatment is a variable calculated at a specific point of follow-up (after two years), the adjustment of the multivariate model for its study was performed by means of generalized linear models using the Stata *glm* command.

### Data availability

All relevant data are within the paper and its Supporting Information files. The datasets generated and/or analysed during the current study are not publicly available due to the confidential nature of the clinical data but are available from the corresponding author on reasonable request.

## Electronic supplementary material

Supplementary Figures
