# Supplementary material for: Vasoactive intestinal peptide gene polymorphisms, associated with its serum levels, predict treatment requirements in early rheumatoid arthritis
Source: Sci Rep. 2018 Feb 1;8:2035. doi: 10.1038/s41598-018-20400-6 (PMC5794878; doi:10.1038/s41598-018-20400-6)
Supplement: Supplementary file 1 — Supplementary Figures [file 41598_2018_20400_MOESM1_ESM.pdf]

Vasoactive Intestinal Peptide gene polymorphisms, associated with its serum levels, predict treatment requirements in early rheumatoid arthritis

Iria V. Seoane, Carmen Martínez, Rosario García-Vicuña, Ana María Ortíz, Yasmina Juarranz, Vanessa C. Talayero, Isidoro González-Álvaro, Rosa P. Gomariz, and Amalia Lamana

Supplementary Table 1. Baseline characteristics of PEARL subpopulation used for the sequencing screening.

	High VIP levels (n=11)	Low VIP levels (n=9)	Total (n=20)	*p*
**Female gender (%)**	8 (73)	5 (56)	13 (65)	0.37
**Age**	58 [49 – 69]	46 [41 – 54]	54 [46 – 64]	0.04
**Diagnosis RA / UA (%)**	7 (64) / 4 (36)	7 (78)/2 (22)	14 (70)/ 6 (30)	0.43
**Positive RF (%)**	3 (27)	4 (44)	7 (35)	0.37
**Positive ACPA (%)**	1 (9)	5 (56)	6 (30)	0.05
**Ever smoker (%)**	4 (40)	4 (50)	8 (45)	0.80
**DAS28 (0-10)**	5.7 [4.4 – 6.8]	4.6 [4.4 – 4.8]	4.8 [4.4 – 6.4]	0.19
**HUPI**	9.5 [7 – 11.5]	7 [6.5 – 8]	8 [6.5 – 11.5]	0.41

Data are shown as the median and the interquartile range or as the percentage. RA: rheumatoid arthritis; UA: undifferentiated arthritis; RF: rheumatoid factor; ACPA: anti-citrullinated peptide antibodies; DAS28: 28-joint count Disease Activity Score; HUPI: Hospital Universitario La Princesa Index for disease activity; HAQ: health assessment questionnaire; VIP: vasoactive intestinal peptide.

Supplementary Table 2. Sequences of the primers used for sequencing *VIP* gene and length of generated amplicons.

AMPLICON	LENGTH (bp)	PRIMER SEQUENCE FORWARD (5′-M13-PRIMER-3′)	PRIMER SEQUENCE REVERSE (5′-M13-PRIMER-3′)
**Amplicon 1**	653	5′TGTAAAACGACGGCCAGTTCAGAGCTGTCAACTGGGAAACAA-3′	5′CAGGAAACAGCTATGACCTGGCACAATTCCCAATGCAC-3′
**Amplicon 2**	751	5′TGTAAAACGACGGCCAGTTGGTTTGAGTATGTGTGTGTGTTGGG-3′	5′CAGGAAACAGCTATGACCGGCCATGGGATGAATAGGGC-3′
**Amplicon 3**	523	5′TGTAAAACGACGGCCAGTTGAAGGAATCCCAATGGCCC-3′	5′CAGGAAACAGCTATGACCGTTCCAGAGGCAGGAGGGAAA-3′
**Amplicon 4**	393	5′TGTAAAACGACGGCCAGTGAGCACGACTGGGCGAGGTA-3′	5′CAGGAAACAGCTATGACCCATACGAAATAGGAAAAAAAAAGCA-3′
**Amplicon 5**	496	5′TGTAAAACGACGGCCAGTTGTAAGATGCTTTACCCTGCTTGCTC-3′	5′CAGGAAACAGCTATGACCACATTACTTTTTCCTCTTTCAATTGTAGA-3′
**Amplicon 6**	685	5′TGTAAAACGACGGCCAGTTGCTTTTTTTTTCCTATTTCGTATG-3′	5′CAGGAAACAGCTATGACCCAGTGACAGGTGCTTGAATAAAAA-3′
**Amplicon 7**	632	5′TGTAAAACGACGGCCAGTGCCAACCATGTGCCCAGACA-3′	5′CAGGAAACAGCTATGACCGAATAAAAAGAAAGTGAAAGCCTCC-3′
**Amplicon 8**	598	5′TGTAAAACGACGGCCAGTATGTGTTGAGTGAGAGGTGTTTGT-3′	5′CAGGAAACAGCTATGACCCCCGTAAACTGAAAGGAAAATTC-3′
**Amplicon 9**	787	5′TGTAAAACGACGGCCAGTGGAGGCTTTCACTTTCTTTTTATTC-3′	5′CAGGAAACAGCTATGACCTATCTTTCAGTGATTTGGGAGTTG-3′
**Amplicon 10**	746	5′TGTAAAACGACGGCCAGTGAATTTTCCTTTCAGTTTACGGG-3′	5′CAGGAAACAGCTATGACCCCTGTTCCAAACATCTCTGAAGAACA-3′
**Amplicon 11**	580	5′TGTAAAACGACGGCCAGTCAACTCCCAAATCACTGAAAGATA-3′	5′CAGGAAACAGCTATGACCTTCTTTGGTGTAATTTACTATGCCAA-3′
**Amplicon 12**	510	5′TGTAAAACGACGGCCAGTTGTTCTTCAGAGATGTTTGGAACAGG-3′	5′CAGGAAACAGCTATGACCCTTGATCAGGTTCATTTGCTCCCT-3′
**Amplicon 13**	756	5′TGTAAAACGACGGCCAGTTTGGCATAGTAAATTACACCAAAGAA-3′	5′CAGGAAACAGCTATGACCCAATGATGGAAGAGTTGCCAAGGA-3′
**Amplicon 14**	624	5′TGTAAAACGACGGCCAGTATGGCTTCATTCATCCTGATTTA-3′	5′CAGGAAACAGCTATGACCCAGGCATTTCTAGGAGCACATTTCA-3′
**Amplicon 15**	744	5′TGTAAAACGACGGCCAGTCAATGATGGAAGAGTTGCCAAGGA-3′	5′CAGGAAACAGCTATGACCGAAAGTTGACCCAAGAGTTTACTGA-3′
**Amplicon 16**	530	5′TGTAAAACGACGGCCAGTTGAAATGTGCTCCTAGAAATGCCTG-3′	5′CAGGAAACAGCTATGACCTCCATGGGCTTGATAGATGTTATTGA-3′
**Amplicon 17**	641	5′TGTAAAACGACGGCCAGTTCAGTAAACTCTTGGGTCAACTTTC-3′	5′CAGGAAACAGCTATGACCTTTTGGCATGGCCTGGGACT-3′
**Amplicon 18**	651	5′TGTAAAACGACGGCCAGTTCAATAACATCTATCAAGCCCATGGA-3′	5′CAGGAAACAGCTATGACCTGACGTTTGACTGGTACAGGGTCTTC-3′
**Amplicon 19**	569	5′TGTAAAACGACGGCCAGTCAGACAAGATGACCTCTTTGCCCA-3′	5′CAGGAAACAGCTATGACCTCCCTATACTTAATTGAGGAGCTAACA-3′
**Amplicon 20**	742	5′TGTAAAACGACGGCCAGTGAAGACCCTGTACCAGTCAAACGTCA-3′	5′CAGGAAACAGCTATGACCTGGCAAAGAGGTCATCTTGTCTG-3′
**Amplicon 21**	683	5′TGTAAAACGACGGCCAGTCGGTGGTGTGGCCCTAGACA-3′	5′CAGGAAACAGCTATGACCCTCCAAAGGTCTTTTTCATCATTT-3′
**Amplicon 22**	742	5′TGTAAAACGACGGCCAGTCAGACAAGATGACCTCTTTGCCCA-3′	5′CAGGAAACAGCTATGACCTCCCTATACTTAATTGAGGAGCTAACA-3′
**Amplicon 23**	603	5′TGTAAAACGACGGCCAGTAAATGATGAAAAAGACCTTTGGAG- 3′	5′CAGGAAACAGCTATGACCCAAACTTACAGGTAAAACAAGCTAAATTT- 3′
**Amplicon 24**	759	5′TGTAAAACGACGGCCAGTTGTTAGCTCCTCAATTAAGTATAGGGA-3′	5′CAGGAAACAGCTATGACCGGGTAATTAGTGGACAAAAGCAGT-3′
**Amplicon 25**	408	5′TGTAAAACGACGGCCAGTAGCCAGATTCCAATCCCTAACTA-3′	5′CAGGAAACAGCTATGACCACCATTTACAAAGCACAGTGTGAT-3′
**Amplicon 26**	498	5′TGTAAAACGACGGCCAGTACTGCTTTTGTCCACTAATTACCC-3′	5′CAGGAAACAGCTATGACCTCTCTTTGTCCCTTGTTAACAGC-3′
**Amplicon 27**	788	5′TGTAAAACGACGGCCAGTATCACACTGTGCTTTGTAAATGGT-3′	5′CAGGAAACAGCTATGACCATACAGCATATGAAATTGCAGGC-3′
**Amplicon 28**	537	5′TGTAAAACGACGGCCAGTGCTGTTAACAAGGGACAAAGAGA-3′	5′CAGGAAACAGCTATGACCCAGCATTCCATCTGCTTAACATTCAA-3′
**Amplicon 29**	681	5′TGTAAAACGACGGCCAGTGCCTGCAATTTCATATGCTGTAT-3′	5′CAGGAAACAGCTATGACCTGAGGGATGAGCTGGAAGGG-3′

Supplementary Table 3. Single Nucleotide Polymorphisms: Name, Location and Minor Allele Frequencies.

				Sequenced Screening population		Genotyped EA population	
Name	**Position (GRCh38.p2)**	**Gene region**	**Alleles**	**High VIP levels (n=11)**	**Low VIP levels (n=9)**	**n**	**MAF**
rs12213214	152749651	Promoter	C:A	1 CA (9%)	2 CA (22%)	506	**0.163**
rs60946248	152750060	Promoter	C:T	0 (0%)	1 CT (11%)	475	**0.001**
rs140023105	152750921	5'UTR Exon 1	A:G	0 (0%)	1 AG (11%)	505	**0.001**
rs35643203	152751298	Intron 1	C:T	0 (0%)	3 CT (33%)	540	**0.065**
rs3799142	152751561	Intron 1	G:A	1 TC (9%)	1 TC (11%)	508	**0.158**
rs7764067	152753064	Intron 2	A:T	1 AT (9%)	2 AT (22%)	502	**0.166**
rs3823082	152753187	Intron 2	G:A	1 GA (9%)	3 GA (33%)	541	**0.24**
rs185451870	152753597	Intron2	A:G	0 AG (0%)	1 AG (11%)	483	**0.001**
rs12201173	152753941	Intron2	T:C	1 TC (9%)	1 TC (11%)	510	**0.159**
rs71575932	152754709	Intron3	A:G	0 AG (0%)	3 AG (33%)	539	**0.073**
rs12201030	152755654	Intron4	A:G	2 AG (18%)	1 AG (11%)	446	**0.111**
rs7755568	152755820	Intron4	T:A	0 TA (0%)	2 TA (22%) 1 AA (11%)	461	**0.06**
rs12201140	152755821	Intron4	A:T	0 AT (0%)	1 AT (11%)	461	**0.143**
rs74760293	152756106	Intron4	T:C	0 TC (0%)	1 TC (11%)	478	**0.004**
rs149081483	152757365	Intron6	T:G	O TG (0%)	1 TG (12%)	466	**0.001**
rs688136	152758926	UTR 3' Exon7	T:C	2 TC (18%) 2 CC (18%)	4 TC (44%)	503	**0.348**

GRCh38.p2: Genome Reference Consortium Human Build 38 patch release 2;UTR: untranslated region; EA: early arthritis; MAF: minor allele frequency.

Supplementary Table 4. Polymorphisms associated to VIP serum levels during the follow-up of patients with early arthritis in a bivariate analysis.

		Discovery Population		Validation Population		Meta-Analysis	
**SNP**	**Alleles**	**β coeff ± SE**	***p***	**β coeff ± SE**	***p***	**β coeff ± SE**	***p***
	CC	Ref.		Ref.		Ref.	
**rs12213214**	CA	-45± 36.0	0.215	18± 16.2	0.261	-3± 17.1	0.841
	AA	112± 94.9	0.239	-14± 28.9	0.631	10± 34.1	0.772
	CC	Ref.		Ref.		Ref.	
**rs35643203**	CT	-110± 48.8	0.024	12± 19.8	0.56	-25± 21.7	0.255
	TT	-24± 147.5	0.872	121± 81.4	0.137	57± 79.6	0.473
	GG	Ref.		Ref.		Ref.	
**rs3823082**	GA	-58± 31.3	0.065	5± 14.5	0.708	-18± 15.1	0.244
	AA	-32± 60.6	0.597	35± 24.2	0.152	13± 26.5	0.636
	AA	Ref.		Ref.		Ref.	
**rs12201030**	AG	-6± 44.6	0.893	-3± 18.0	0.877	-5± 19.8	0.786
	GG	-	-	-27± 43.7	0.539	-31± 58.5	0.597
	TT	Ref.		Ref.		Ref.	
**rs7755568**	TA	-109± 59.7	0.068	-4± 20.5	0.828	-35± 24.0	0.149
	AA	-178± 98.7	0.071	124± 77.2	0.109	-68± 61.6	0.27
	AA	Ref.		Ref.		Ref.	
**rs12201140**	AT	-48± 39.4	0.223	-15± 15.0	0.307	-26± 16.7	0.115
	TT	-16± 130.5	0.904	-96± 77.4	0.216	-49± 71.2	0.487
	TT	Ref.		Ref.		Ref.	
**rs688136**	TC	3± 31.0	0.921	1± 14.0	0.933	2± 15.0	0.873
	CC	57± 49.8	0.251	70± 23.1	0.002	65± 24.5	0.008

The analysis was performed with data from consecutive visits corresponding to 91 patients for the discovery population, 131 for the validation population and the sum of all for the meta-analysis. Included patients have, at least, two visits along the follow-up. Signification was established be means of generalized estimating equations nested by patient and visit. Ref.: reference value; β coeff: beta coefficient.

Supplementary Table 5. Polymorphisms associated to VIP serum levels during the follow-up of patients with early arthritis in a multivariate analysis.

	**Discovery Population**				**Validation Population**			
	Model 1		***Model 2***		***Model 1***		***Model 2***	
	**β coeff ± SE**	***p***	**β coeff ± SE**	***p***	**β coeff ± SE**	***p***	**β coeff ± SE**	***p***
**rs688136**								
TT	Ref.		-	-	Ref.		-	-
TC	5 ± 34.9	0.887			-10 ± 14.2	0.477		
CC	142 ± 66.5	0.033			66 ± 24.4	0.007		
**rs35643203**								
CC	Ref.		-	-	Ref.		-	-
CT	-123 ± 62.7	0.05			-5 ± 19.8	0.795		
TT	-189 ± 171.2	0.269			129 ± 72.8	0.075		
**rs12201140**								
AA	Ref.		-	-	Ref.		-	-
AT	-73 ± 39.9	0.068			-20 ± 14.9	0.187		
TT	-181 ± 137.7	0.188			-92 ± 72.2	0.202		
**Interaction Score**								
CT or TT and no other minor alleles	-	-	Ref.		-	-	Ref.	
CT or TT and other minor alleles			-54 ± 25.7	0.037			-26 ± 13.9	0.066
CC and no other minor alleles			173 ± 69.7	0.013			67 ± 34.3	0.050
CC and other minor alleles			-45 ± 50.1	0.369			60 ± 27.6	0.029
**Onset Age**								
<45	Ref.		Ref.		Ref.		Ref.	
45 - 65	96 ± 38.6	0.013	63 ± 30	0.032	-1 ± 16.5	0.976	10 ± 15.9	0.530
> 65	77 ± 44.6	0.083	62 ± 33	0.060	-5 ± 17.5	0.761	15 ± 17.2	0.380
**TNF blockers**								
No	Ref.		Ref.		Ref.		Ref.	
Yes	170 ± 66.9	0.011	142 ± 54	0.008	12 ± 33.3	0.712	10 ± 32.5	0.752

The longitudinal analysis was performed with data from consecutive visits corresponding to 91 patients for the discovery population and 131 for the validation population. Included patients have, at least, two visits along the follow-up. Signification was established be means of generalized estimating equations nested by patient and visit. Multivariate model fitted by a backward-stepwise selection. Model 1 built-in including independent polymorphisms and Model 2 including the Interaction Score. Ref.: reference value; β coeff: beta coefficient; SE: standard error; TNF: tumor necrosis factor.

Supplementary Table 6. Association of genotype combinations of the Interaction Score and the Intensity of Treatment in patients with early arthritis. Multivariate analysis.

	**Discovery Population**		**Validation Population**	
	**β coeff ± SE**	***p***	**β coeff ± SE**	***p***
**Interaction Score**				
CT or TT and no other minor alleles	Ref.		Ref.	
CT or TT and other minor alleles	0.10 ± 0.15	0.509	-0.44 ± 0.17	0.008
CC and no other minor alleles	-0.54 ± 0.41	0.183	-1.11 ± 0.43	0.009
CC and other minor alleles	0.15 ± 0.34	0.663	0.22 ± 0.33	0.496
**Gender**				
Male	Ref.		Ref.	
Female	0.46 ± 0.19	0.018	0.21 ± 0.20	0.287
**OnsetAge**				
< 45	Ref.		Ref.	
45 - 65	-0.04 ± 0.19	0.846	0.17 ± 0.20	0.406
> 65	-0.12 ± 0.21	0.570	0.01 ± 0.21	0.951
**Diagnosis**				
RA	Ref.		Ref.	
UA	-0.53 ± 0.20	0.007	-0.88 ± 0.19	0.000
**ACPA**				
Negative	Ref.		Ref.	
Positive	0.27 ± 0.17	0.109	-0.18 ± 0.19	0.338
**Physician**				
0	Ref.		Ref.	
1	0.01 ± 0.32	0.969	-0.02 ± 0.34	0.948
2	0.24 ± 0.26	0.361	0.30 ± 0.30	0.316
3	-0.78 ± 0.52	0.133	-0.65 ± 0.39	0.093
4	-0.14 ± 0.23	0.550	-0.16 ± 0.28	0.561
5	0.40 ± 0.24	0.100	-0.23 ± 0.38	0.550
6	0.27 ± 0.25	0.281	0.39 ± 0.24	0.059

Association analysis performed with data corresponding to 91 patients for the discovery population and 131 for the validation population. Signification was established be means of generalized estimating equations. Final multivariate model, fitted by a backward-stepwise selection, was adjusted by gender, age of onset, final diagnostic, presence of anti-citrullinated peptide antibodies and the physician who prescribed medication. Ref.: reference value; β coeff: beta coefficient; SE: standard error.
